# Eight genome sequences of bacterial, environmental isolates from Canada Glacier, Antarctica

**DOI:** 10.1128/mra.01130-23

**Published:** 2024-07-11

**Authors:** Heidi J. Smith, Markus Dieser, Christine M. Foreman

**Affiliations:** 1 Center for Biofilm Engineering, Montana State University, Bozeman, Montana, USA; 2 Department of Microbiology and Cell Biology, Montana State University, Bozeman, Montana, USA; 3 Department of Chemical & Biological Engineering, Montana State University, Bozeman, Montana, USA; SUNY College of Environmental Science and Forestry, Syracuse, New York, USA

**Keywords:** Antarctica, glacier, cryoconite hole, stream

## Abstract

Sediments in cryoconite holes and meltwater streams in the McMurdo Dry Valleys, Antarctica, provide both substrates and conditions that support life in an arid polar desert. Here, we report the genomic sequences of eight environmental, bacterial isolates from Canada Glacier cryoconite holes and stream. These isolates span three major phyla.

## ANNOUNCEMENT

In the barren polar desert of the McMurdo Dry Valleys, Antarctica, meltwater-driven supraglacial depressions (i.e., cryoconite holes) and streams such as those in the Canada Glacier basin are among the top refuges for microorganisms (1 – 6). Cryoconite holes are cylindrical, water-filled depressions on glacial surfaces formed by the deposition and accumulation of aeolian material. The Canada Stream is an ephemeral stream, fed by runoff from the Canada Glacier.

While microbes thrive in cryoconite holes and the stream channel (7 – 10), rapid response and adaptation are required to withstand frequent disturbances, freezing, desiccation, and transition to quiescence. Our goal is to understand the genetic traits linked to persistence under the multitude of environmental stressors in these systems.

Cryoconite and stream water samples from the Canada Glacier (77°37′S, 162°59′E) were collected in December 2009 and are described elsewhere (9). Bacteria were isolated aerobically on R2A agar plates at 4°C. The cetyltrimethylammonium bromide procedure was used for extracting DNA from bacterial isolates grown to late exponential phase in R2A broth at 4°C while shaking at 150 rpm (11). Standard quality shotgun libraries for bacterial strains CAN_C2, CAN_C3, CAN_C7, CAN_S1, CAN_S2, CAN_S4, and CAN_S7 were sequenced on the Illumina NovaSeq S4 platform (2 × 151 bp paired-end reads) (12). Libraries were prepared on the PerkinElmer Sciclone NGS robotic liquid handling workstation using the Kapa Biosystems Library Kit. DNA was sheared to 459 bp using a Covaris LE220-focused ultrasonicator. DNA fragments were size selected by double solid phase reversible immobilization (SPRI). Fragments were end repaired, A tailed, and ligated with Illumina compatible sequencing adaptors. Raw Illumina sequences were quality filtered using BBTools v38.95 (QV ≥20; length ≥100 bases) (13) per JGI SOP 1061 and assembled with SPAdes (≥version v3.14.1; –phred-offset 33 –cov-cutoff auto -t 16 m 64 –careful -k 25,55,95) (14). Contigs with a length <1 kb (BBTools reformat.sh: minlength = 1,000 ow = t) were discarded.

For improved high-quality draft genomes, Pacbio SMRTbell libraries for bacterial strain CAN_C5 were sequenced on Pacific Biosciences (PacBio) Sequel platform (15). PacBio sequencing generated 889,350 reads with an average length of 8,908 bp. DNA was sheared around 10 kb using Megaruptor 3 (Diagenode). Sheared DNA was treated with exonuclease, DNA repair enzyme mix, end-repair/A-tailing mix, and ligated with barcoded overhang adapters using SMRTbell Express Template Prep Kit 2.0 (PacBio). Libraries were purified with AMPure PB Beads (PacBio) and bound to Sequel II polymerase 2.0 using the Sequel II Binding Kit 2.0. Libraries were sequenced using tbd-sample-dependent sequencing primers, 8M v1 SMRT cells, and Version 2.0 sequencing chemistry with 1 × 900 sequencing movie run times. Reads >5 kb were assembled with hierarchical genome assembly process (HGAP) [smrtlink/8.0.0.80529, HGAP 4 (1.0)] using default settings (16).

CRISPR elements were identified using the program CRT (CRISPR Recognition Tool) (17) or PILER-CR (18). All genomes were annotated in the Integrated Microbial Genomes (IMG) database (≥IMG Annotation Pipeline v.5.0.19) (19). Genome completeness was estimated using checkM (20). The genome-sequence-acquired 16S ribosomal RNA gene was queried in command line against the SILVA database 138.1 with blastn, from BLAST+ 2.13.0 for taxonomic classification (21). Sequence details are given in Table 1.

**TABLE 1:** Summary of genome characteristics^*a*^

Platform	Organism (% match to genus) Accession number	Coverage (×)	# Contigs	L50/N50 (bp)	Size (bp)	GC (%)	# Genes	# C
Illumina	*Cryobacterium* sp. CAN_C2 (99.5) JADOUX000000000 SRR13292036	419.2	58	8/149,434	3,600,773	64.66	3,541	–^*b*^
Illumina	*Cryobacterium* sp. CAN_C3 (99.4) JAXCHQ000000000 SRR25764242	394.8	60	9/149,434	3,604,084	64.65	3,519	–
PACBIO	*Arthrobacter* sp. CAN_C5 (99.6) JAGGMZ000000000 SRR26356106	250.1	1	1/3,860,921	3,860,921	64.15	3,796	–
Illumina	*Mycetocola* sp. CAN_C7 (97.8) JADOUY000000000 SRR13164828	374.4	20	3/556,454	3,420,744	65.49	3,308	–
Illumina	*Janthinobacterium* sp. CAN_S1 (99.7) JADOUZ000000000 SRR13164876	252.0	72	10/151,624	4,870,004	60.87	4,452	–
Illumina	*Flavobacterium* sp. CAN_S2 (99.6) JADOVA000000000 SRR13164877	197.8	17	2/469,638	3,844,435	34.22	3,572	2
Illumina	*Salinibacterium* sp. CAN_S4 (99.6) JADOVB000000000 SRR13164888	384.9	11	2/547,050	3,068,493	65.19	3,035	1
Illumina	*Janthinobacterium* sp. CAN_S7 (98.4) JBANDI000000000 SRR25764251	328.9	68	12/134,133	5,015,770	60.65	4,645	–

^*a*^ C = CRISPR.

^*b*^ –, none.

## Data Availability

The JGI genome project (ID: 505037) is available from the IMG database. The genome sequences have been submitted to GenBank under the accession numbers listed in Table 1.
